# Associations among socioeconomic status, multimorbidity of non-communicable diseases, and the risk of household catastrophic health expenditure in China: a population-based cohort study

**DOI:** 10.1186/s12913-023-09391-x

**Published:** 2023-04-26

**Authors:** Yaping Wang, Min Du, Chenyuan Qin, Qiao Liu, Wenxin Yan, Wannian Liang, Min Liu, Jue Liu

**Affiliations:** 1 Department of Epidemiology and Biostatistics, School of Public Health, Peking University, Beijing, China; 2 Vanke School of Public Health, Tsinghua University, Beijing, China; 3 Institute for Global Health and Development, Peking University, Beijing, China; 4 Key Laboratory of Reproductive Health, National Health and Family Planning Commission of the People’s Republic of China, Beijing, China; 5 Key Laboratory of Epidemiology of Major Diseases (Peking University), Ministry of Education, Beijing, China

**Keywords:** Multimorbidity, Catastrophic health expenditure, Universal health coverage, Aging population

## Abstract

**Background:**

Multimorbidity of non-communicable diseases (NCDs) is increasingly prevalent among older adults around the world, leading a higher risk of household catastrophic health expenditure (CHE). As current powerful evidence was insufficient, we aimed to estimate the association between multimorbidity of NCDs and the risk of CHE in China.

**Methods:**

We designed a cohort study using data investigated in 2011–2018 from the China Health and Retirement Longitudinal Study, which is a nationally-representative study covering 150 counties of 28 provinces in China. We used mean ± standard deviation (SD) and frequencies and percentages to describe baseline characteristics. Person χ2 test was employed to compare the differences of baseline characteristics between households with and without multimorbidity. Lorenz curve and concentration index were used to measure the socioeconomic inequalities of CHE incidence. Cox proportional hazards models were applied to estimate adjusted hazard ratios (aHRs) and 95% confidence intervals (CIs) for the association between multimorbidity and CHE.

**Results:**

Among 17,708 participants, 17,182 individuals were included for the descriptive analysis of the prevalence of multimorbidity in 2011, and 13,299 individuals (8029 households) met inclusion criteria and were included in the final analysis with a median of 83 (interquartile range: 25–84) person-months of follow-up. 45.1% (7752/17,182) individuals and 56.9% (4571/8029) households had multimorbidity at baseline. Participants with higher family economic level (aOR = 0.91, 95% CI: 0.86–0.97) had lower multimorbidity prevalence than those with lowest family economic level. 82.1% of participants with multimorbidity did not make use of outpatient care. The CHE incidence was more concentrated among participants with higher socioeconomic status (SES) with a concentration index of 0.059. The risk of CHE was 19% (aHR = 1.19, 95% CI: 1.16–1.22) higher for each additional NCD.

**Conclusions:**

Approximately half of middle-aged and older adults in China had multimorbidity, causing a 19% higher risk of CHE for each additional NCD. Early interventions for preventing multimorbidity among people with low SES could be intensified to protect older adults from financial hardship. In addition, concerted efforts are needed to increase patients’ rational healthcare utilization and strengthen current medical security for people with high SES to reduce economic disparities in CHE.

**Supplementary Information:**

The online version contains supplementary material available at 10.1186/s12913-023-09391-x.

## Background

In 2015, 17 goals for ending poverty, protecting the planet and improving the health of everyone everywhere, which are widely known as the Sustainable Development Goals (SDGs), were adopted by the United Nations [1]. Universal health coverage (UHC) is a main content of SDG target 3.8, which aims for all people to have access to the health services they need, when and where they need them, without financial hardship [2]. According to the World Health Organization (WHO), monitoring progress towards UHC should focus on 2 parts: how many people can receive essential quality health services and how much household income people spend on health. For the latter, catastrophic health expenditure (CHE), which is defined as out-of-pocket health expenditure exceeding a specific proportion of household financial capability, is a broadly used indicator despite inconsistent thresholds and denominators among studies [3].

To promote process towards UHC, the Chinese government introduced a series of policies. In the early 2000s, China developed three insurance programs: Urban Employee Basic Medical Insurance (UEBMI) for the employed urban population, the New Rural Cooperative Medical Scheme (NRCMS) for rural residents, and Urban Resident Basic Medical Insurance (URBMI) for urban residents who are unemployed or self-employed. In April 2009, China launched a major health care reform that included increasing financial investment to expand insurance coverage and intensify the development of basic medical institutions [4]. Since 2013, catastrophic medical insurance, which was introduced in 2012 and focused on providing supplementary coverage for patients with URBMI and the NRCMS, has covered more than 1 billion people, and benefited over 11 million people [5]. In 2016, President Xi Jinping announced *Healthy China 2030*, the country’s long-term health sector strategy for a healthy population in all aspects [6].

As early as2011, almost all people in China were insured by one of the abovementioned three social health insurance programs. After10 years of health care reform, China made substantial progress towards UHC, with a steady downward trend in the proportion of households with CHE during 2010–2018. [7] However, as non-communicable diseases (NCDs) are increasingly prevalent in China, additional medical needs and health expenditures are needed [8]. Using data from a national population-based screening project, Lu et al. [9] found that 44.7% of 1,738,886 participants in China had hypertension, only half of them were aware of their diagnosis, and only 7.2% had achieved control. Another nationally representative survey revealed that the estimated prevalence of diagnosed diabetes in China was 10.9% (95% confidence interval (CI): 10.4-11.5%) in 2013 and 12.4% (95% CI: 11.8-13.0%) in 2018, absolute increase of 1.4% (95% CI: 0.7-2.2%), and only 32.9% (95% CI: 30.9-34.8%) of individuals reported being treated [10]. Hence, further-integrated efforts need be made to consolidate current achievements and promote UHC.

With ageing of population, the prevalence of multimorbidity (defined as the coexistence of 2 or more NCDs in one person) is increasing among older adults. In a systematic review, the median prevalence of multimorbidity was 30.7% among all people in China, and it was about half of the prevalence among older adults [11–13]. Such a severe disease burden directly triggers a high amount of medical expenditure and CHE incidence. A national study conducted in China found that for each additional chronic disease, the risk of CHE increased by 39% (odds ratio (OR) = 1.39, 95% CI: 1.31–1.47). [14] Similarly, in a Chinese population-based panel study, the likelihood of CHE events increased with the number of NCDs (OR = 1.29, 95% CI: 1.26–1.32). [15] Therefore, the burden of multimorbidity in the older population is an urgent point that needs to be considered to reduce CHE, and to solve this problem needs more evidence about the effect of multimorbidity on CHE. However, current related results were obtained from cross-sectional studies with limited data, which cannot provide stronger facts about the association between multimorbidity and CHE or the long-term impacts of multimorbidity on CHE. Moreover, they did not analyze the socioeconomic inequalities of CHE incidence [14–17].

This study aimed to estimate the association of multimorbidity with CHE incidence among middle-aged and older adults through a population-based cohort study design using all wave data (2011, 2013, 2015, and 2018) from the China Health and Retirement Longitudinal Study (CHARLS).

## Methods

### Study design and participants

CHARLS is a nationally representative survey implemented by the National School of Development of Peking University of the middle-aged and older population of China, including individuals aged ≥ 45 years [18]. Considering the sample lost during follow-up and the long period of investigation, CHARLS included some people aged < 45 years if they met the inclusion criteria as a reserved sample for future interviews. The baseline survey was conducted between June 2011 and March 2012 and ultimately included 17,708 eligible participants from 150 counties of 28 provinces in China. CHARLS respondents were followed up every 2 years via face-to-face interviews with a structured questionnaire that included individual and family components. In the family component of the questionnaire, CHARLS selected one individual randomly from each household as the main respondent. Details about the design of CHARLS have been published elsewhere [18]. All data were openly available from the official CHARLS website (http://charls.pku.edu.cn/). The Biomedical Ethics Review Committee of Peking University approved CHARLS (ethical approval number IRB00001052-11015), and all subjects provided written informed consent before enrolment.

**Fig. 1:**
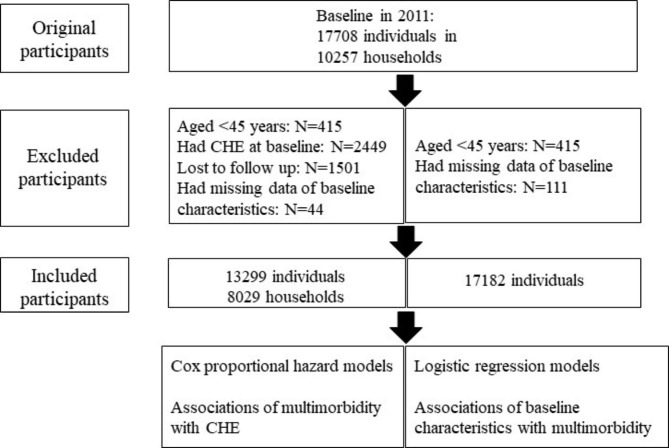
Flowchart of the study participants. Note: CHE: catastrophic health expenditure

We designed a population-based cohort study using data from all waves (2011, 2013, 2015, and 2018) of CHARLS. The exposure at baseline was the number of NCDs, and the outcome was the onset of CHE. The participants were followed up until they had CHE onset or the interview ended. Among 17,708 participants selected in baseline survey, we excluded those who aged < 45 years (n = 415), had CHE at baseline (n = 2449), were lost to follow-up (n = 1501), and had missing data on baseline characteristics (n = 44). In addition, 5270 participants who were not the main respondents of households were excluded from the main household-related analysis. Finally, a total of 13,299 individuals and 8029 households (main respondents) were included in this study (Fig. 1). After excluding those aged < 45 years (N = 415) and those who had missing data on baseline characteristics (N = 111), 17,182 individuals were included in the analysis of the association between baseline characteristics and multimorbidity.

### Measures of multimorbidity

In this study, we described multimorbidity as the cooccurrence of at least 2 chronic NCDs in the same person. We ascertained 14 self-reported diagnosed chronic disorders to measure multimorbidity by asking “Have you been diagnosed with the following conditions by a doctor: hypertension, dyslipidemia, diabetes or high blood sugar, cancer or malignant tumor, chronic lung diseases, liver disease, heart diseases, stroke, kidney disease, stomach or other digestive disease, psychiatric problems, memory-related diseases, arthritis or rheumatism, or asthma?”. We counted the number of NCDs from the answers of participants, and divided participants into 2 groups: those with multimorbidity (> 1 NCD) and those without multimorbidity (0 or 1 NCD).

### Measures of household catastrophic health expenditure

Participants in CHARLS were asked about their use of outpatient care during the past month, inpatient care during the past year, and self-medication during the past month. The out-of-pocket (OOP) expenditure for each kind of medical service was reported by individuals. We multiplied the monthly spending by 12 to calculate the yearly OOP expenditure of outpatient care and self-medication for each participant. Then we calculated the self-medical OOP expenditure for each participant as the sum of the annual OOP expenditures of outpatient care, inpatient care, and self-medication and added it to their spouses’ as household medical OOP expenditure. In this study, a household with CHE was defined as having a household medical OOP expenditure exceeding 40% of the household’s capacity to pay (defined as the total household expenditure minus household food expenditure). [3] The annual household food expenditure was calculated as the weekly meal expenses divided by 7 and then multiplied by 365, and the annual total household expenditure was calculated as the sum of monthly basic expenditures (daily used commodities and necessities, communication, transportation, fuel, and so on) multiplied by 12 plus yearly special expenditures (clothing, travelling, education, furniture, and so on).

### Covariates

To control for potential confounders in the association between multimorbidity and CHE, we included the following variables as covariates: gender (male, female), age group (45–54, 55–64, ≥ 65 years), marital status (married, other), education (illiterate/semiliterate, primary school, middle school, high school and above), insurance (no insurance, UEBMI, URBMI, NRCMS, other), outpatient services (yes, no), inpatient services (yes, no), family size (1–2, 3–4, ≥ 5), residence (urban, rural), family economic level (four classes), and socioeconomic development level (four classes).

Outpatient and inpatient services were identified by the questions “Have you visited a hospital or clinic center for outpatient care in the last month?” and “Have you received inpatient care in the past year?”. The family economic level was classified by the quartiles of household annual income (lowest: <4000 yuan; lower: 4000–16,799 yuan; higher: 16,800–38,399 yuan; highest: ≥38,400 yuan). The socioeconomic development level was identified by the quartiles of 2011 per capita gross regional product (GRP, lowest: <27,250 yuan; lower: 27,250–33,232 yuan; higher: 33,232–48,200 yuan; highest: ≥48,201 yuan), which were obtained from the 2011 China Statistical Yearbook [19].

### Statistical analysis

We described baseline characteristics using means ± standard deviations (SDs) for continuous variables and frequencies and percentages for categorical variables. To compare the distributional differences in the characteristics of household heads with and without multimorbidity, we used the Pearson χ2 test.

The concentration index (C) and Lorenz curve were applied to evaluate the socioeconomic inequalities in CHE incidence. In this study, socioeconomic inequality was defined by dividing annual family income into quartiles (lowest, lower, higher, highest). C (ranging between − 1 and 1) is defined as twice the area between the concentration curve and the line of equality and can be calculated by the following formula:

$$C = \frac{2}{{\mu \times {\rm{cov}}({\rm{h}},{\rm{r}})}}$$

where h refers to the health outcome (CHE), µ is the mean of h, and r denotes the fractional rank of individuals in family income quartiles. If C took a negative value, in this study, it indicated that CHE events are more concentrated among poor people. Conversely, if C was a positive value, it indicated that CHE events were more concentrated among rich people [20]. The Lorenz curve was plotted by the cumulative percentage of CHE incidence (Y-axis) against the cumulative percentage of the population ranked by family income (X-axis).

At the individual level, a logistic regression model was employed to estimate the adjusted odds ratios (aORs) and 95% confidence intervals (CIs) for the association of baseline characteristics (gender, age group, marital status, education, insurance, family size, residence, family income level, and socioeconomic development level) with multimorbidity (with and without). At the household level, we applied univariate and multivariate Cox proportional hazards models to estimate the hazard ratios (HRs) and 95% CIs for the effect of multimorbidity on household CHE. In the multivariate Cox proportional hazards model, we adjusted for all the covariates, including gender, age group, marital status, education, insurance, outpatient services, inpatient services, family size, residence, family economic level, and socioeconomic development level.

To examine the robustness of estimations for the impact of multimorbidity on CHE, we performed three sensitivity analyses. First, we replaced the definition of CHE as household-medical OOP expenditure exceeding 25% of total household expenditure with the WHO definition. Second, we changed the original selected main respondents in households with 2 eligible respondents to the 5270 excluded participants and used the same fully-adjusted model. Third, we used the nighttime light level (divided into four classes according to the quartiles of 2011 province-level mean nighttime light intensity [21]) to indicate socioeconomic development rather than GRP.

To explore the possible different effects of the number of NCDs on CHE incidence among subpopulations, the analysis was stratified by age group, insurance, residence, family economic level, and socioeconomic development level, using the same fully-adjusted model but with the stratified variable removed.

Considering the national representativeness, multistage probability proportional to size (PPS) sampling and nonresponse rate of CHARLS, all data included in the analyses were weighted per the sampling probabilities [22]. All analyses were conducted in R 4.2.1 (R Core Team, Vienna, Austria). A two-side *P* value less than 0.05 was considered to be significant.

## Results

### Baseline individual characteristics and prevalence of multimorbidity

Among all 17,182 participants, the mean (SD) age was 59.4 (9.9) years. A total of 8364 (48.7%) participants were male, 14,948 (87.0%) were married or partnered, and only 1154 (6.7%) participants had no medical insurance. A total of 10,261 (59.7%) participants lived in rural areas, and 10,988 (64.0%) had more than 2 family members (Table 1). Weighted proportions were similar to unweighted results (Table S1).

**Table 1:** Distribution of baseline characteristics for 17,182 participants and the results of Logistic regression models

	Total (N = 17,182)	Without multimorbidity (N = 9430)	With multimorbidity (N = 7752)	aOR (95% CI)	*p value*
**Gender**					
Male	8364 (48.7)	4797 (50.9)	3567 (46.0)	1.00 (ref.)	—
Female	8818 (51.3)	4633 (49.1)	4185 (54.0)	1.16 (1.09, 1.24)	< 0.001*
**Age (years)**					
45 ~ 54	6020 (35.0)	3876 (41.1)	2144 (27.7)	1.00 (ref.)	—
55 ~ 64	6433 (37.4)	3422 (36.3)	3011 (38.8)	1.71 (1.60, 1.82)	< 0.001*
65~	4729 (27.5)	2132 (22.6)	2597 (33.5)	0.96 (0.91, 1.01)	0.156
**Marital status**					
Married	14,948 (87.0)	8322 (88.3)	6626 (85.5)	1.00 (ref.)	—
Other	2234 (13.0)	1108 (11.7)	1126 (14.5)	1.00 (0.90, 1.1)	0.931
**Education**					
Illiterate/semiliterate	7814 (45.5)	4034 (42.8)	3780 (48.8)	1.00 (ref.)	—
Primary school	3617 (21.1)	1951 (20.7)	1666 (21.5)	0.90 (0.83, 0.98)	0.013*
Middle school	3545 (20.6)	2140 (22.7)	1405 (18.1)	1.00 (0.93, 1.07)	0.960
High school and above	2206 (12.8)	1305 (13.8)	901 (11.6)	1.09 (1.01, 1.16)	0.019*
**Insurance**					
None	1154 (6.7)	690 (7.3)	464 (6.0)	1.00 (ref.)	
UEBMI	1892 (11.0)	1000 (10.6)	892 (11.5)	1.49 (1.27, 1.76)	< 0.001*
URBMI	784 (4.6)	358 (3.8)	426 (5.5)	1.81 (1.50, 2.20)	< 0.001*
NRCMS	12,614 (73.4)	7014 (74.4)	5600 (72.2)	1.17 (1.03, 1.33)	0.016*
Other	738 (4.3)	368 (3.9)	370 (4.8)	1.55 (1.27, 1.89)	< 0.001*
**BMI**					
Normal	10,849 (63.1)	6333 (67.2)	4516 (58.3)	1.00 (ref.)	—
Underweight	966 (5.6)	507 (5.4)	459 (5.9)	1.05 (0.92, 1.20)	0.472
Overweight	3892 (22.7)	1995 (21.2)	1897 (24.5)	1.40 (1.29, 1.51)	< 0.001*
Obesity	1475 (8.6)	595 (6.3)	880 (11.4)	2.23 (1.99, 2.50)	< 0.001*
**Family size**					
1 ~ 2	6194 (36.0)	3173 (33.6)	3021 (39.0)	1.00 (ref.)	—
3 ~ 4	5886 (34.3)	34,561 (36.7)	2425 (31.3)	1.00 (0.94, 1.06)	0.935
5~	5102 (29.7)	2796 (29.7)	2306 (29.7)	1.05 (0.99, 1.11)	0.078
**Residence**					
Urban	6921 (40.3)	3775 (40.0)	3146 (40.6)	1.00 (ref.)	—
Rural	10,261 (59.7)	5655 (60.0)	4606 (59.4)	1.02 (0.95, 1.10)	0.565
**Family economic level**					
Lowest	4207 (24.5)	2223 (23.6)	1984 (25.6)	1.00 (ref.)	—
Lower	4315 (25.1)	2258 (23.9)	2057 (26.5)	0.93 (0.86, 1.00)	0.046
Higher	4341 (25.3)	2415 (25.6)	1926 (24.8)	0.91 (0.86, 0.97)	0.003*
Highest	4319 (25.1)	2534 (26.9)	1785 (23.0)	1.04 (0.98, 1.11)	0.197
**Economic development level**					
Lowest	5398 (31.4)	2788 (29.6)	2610 (33.7)	1.00 (ref.)	—
Lower	2924 (17.0)	1608 (17.1)	1316 (17.0)	0.81 (0.76, 0.86)	< 0.001*
Higher	4727 (27.5)	2551 (27.1)	2176 (28.1)	0.96 (0.90, 1.02)	0.169
Highest	4133 (24.1)	2483 (26.3)	1650 (21.3)	0.90 (0.85, 0.97)	0.003*

Notes: aOR: adjusted odds ratio; CI: confidence interval; UEBMI: urban employee basic medical insurance; URBMI: urban resident basic medical insurance; NRCMS: new rural cooperative medical scheme; BMI: body mass index

*p < 0.05

At baseline, among a total of 17,182 participants, 7752 (45.1%) individuals had multimorbidity, and the weighted prevalence of multimorbidity was 44.3%. Logistic regression models revealed that participants with higher family economic level (aOR = 0.91, 95% CI: 0.86–0.97) had lower multimorbidity prevalence than those with lowest family economic level, and participants with lower (aOR = 0.81, 95% CI: 0.76–0.86) and highest (aOR = 0.90, 95% CI: 0.85–0.97) economic development levels were also less likely to have multimorbidity (Table 1).

### Baseline household characteristics in the cohort study

Of the 10,257 households in CHARLS, 8029 households met all the inclusion criteria of this study. A total of 1134 (14.1%) and 505 (6.3%) household heads had outpatient and inpatient care at baseline, respectively. 4850 (60.4%) households were in rural areas, and 5266 (65.6%) households had more than 2 family members (Table 2).

**Table 2:** Distribution of baseline characteristics for 8029 eligible households

Characteristics of household heads	Total (N = 8029)	Without multimorbidity (N = 3458)	With multimorbidity (N = 4571)	*p* value
**Gender**				0.006^*^
Male	3787 (47.2)	1692 (48.9)	2095 (45.8)	
Female	4242 (52.8)	1766 (51.1)	2476 (54.2)	
**Age**				< 0.001^*^
45 ~ 54	2888 (36)	1485 (42.9)	1403 (30.7)	
55 ~ 64	2910 (36.2)	1124 (32.5)	1786 (39.1)	
65~	2231 (27.8)	849 (24.6)	1382 (30.2)	
**Marital status**				< 0.001^*^
Married	6333 (78.9)	2586 (74.8)	3747 (82.0)	
Other	1696 (21.1)	872 (25.2)	824 (18.0)	
**Education**				0.014^*^
Illiterate/semiliterate	3748 (46.7)	1567 (45.3)	2181 (47.7)	
Primary school	1665 (20.7)	698 (20.2)	967 (21.2)	
Middle school	1614 (20.1)	728 (21.1)	886 (19.4)	
High school and above	1002 (12.5)	465 (13.4)	537 (11.7)	
**Insurance**				< 0.001^*^
None	553 (6.9)	275 (8.0)	278 (6.1)	
UEBMI	840 (10.5)	335 (9.7)	505 (11.0)	
URBMI	343 (4.3)	117 (3.4)	226 (4.9)	
NRCMS	5958 (74.2)	2592 (75.0)	3366 (73.6)	
Other	335 (4.2)	139 (4.0)	196 (4.3)	
**Outpatient care**				< 0.001^*^
No	6895 (85.9)	3140 (90.8)	3755 (82.1)	
Yes	1134 (14.1)	318 (9.2)	816 (17.9)	
**Inpatient care**				< 0.001^*^
No	7524 (93.7)	3332 (96.4)	4192 (91.7)	
Yes	505 (6.3)	126 (3.6)	379 (8.3)	
**BMI**				< 0.001^*^
Normal	5128 (63.9)	2345 (67.8)	2783 (60.9)	
Underweight	467 (5.8)	203 (5.9)	264 (5.8)	
Overweight	1744 (21.7)	678 (19.6)	1066 (23.3)	
Obesity	690 (8.6)	232 (6.7)	458 (10.0)	
**Residence**				0.329
Urban	3179 (39.6)	1348 (39.0)	1831 (40.1)	
Rural	4850 (60.4)	2110 (61.0)	2740 (59.9)	
**Family size**				< 0.001^*^
1 ~ 2	2763 (34.4)	1168 (33.8)	1595 (34.9)	
3 ~ 4	2852 (35.5)	1312 (37.9)	1540 (33.7)	
5~	2414 (30.1)	978 (28.3)	1436 (31.4)	
**Family economic level**				0.116
Lowest	1992 (24.8)	872 (25.2)	1120 (24.5)	
Lower	1990 (24.8)	817 (23.6)	1173 (25.7)	
Higher	2035 (25.3)	870 (25.2)	1165 (25.5)	
Highest	2012 (25.1)	899 (26.0)	1113 (24.3)	
**Economic development level**				< 0.001^*^
Lowest	2603 (32.4)	1072 (31)	1531 (33.5)	
Lower	1347 (16.8)	567 (16.4)	780 (17.1)	
Higher	2213 (27.6)	911 (26.3)	1302 (28.5)	
Highest	1866 (23.2)	908 (26.3)	958 (21.0)	

Notes: UEBMI: urban employee basic medical insurance; URBMI: urban resident basic medical insurance; NRCMS: new rural cooperative medical scheme; BMI: body mass index

*p < 0.05

In total 4571 (59.2%) households were defined as with multimorbidity because at least 1 member in this family had more than 1 NCD, and the weighted prevalence of multimorbidity among households was 56.6% (Table S2). 82.1% of household heads with multimorbidity did not make use of outpatient care in the last year (Table 2). Except for residence and family economic level, the distribution of baseline characteristics between households with multimorbidity and those without multimorbidity were significantly different (all *p* value < 0.05; Table 2).

### CHE incidence and its socioeconomic disparities

Of 8029 households, during a median of 83 (interquartile range: 25–84) person-months of follow-up, up to 4260 households had CHE events, with an incidence rate of 8.5 per 1,000 person-months. The cumulative number of CHE events was 1521 (incidence rate: 6.6 per 1,000 person-month) for households without multimorbidity and 2739 (incidence rate: 10.2 per 1,000 person-months) for households with multimorbidity.

**Fig. 2:**
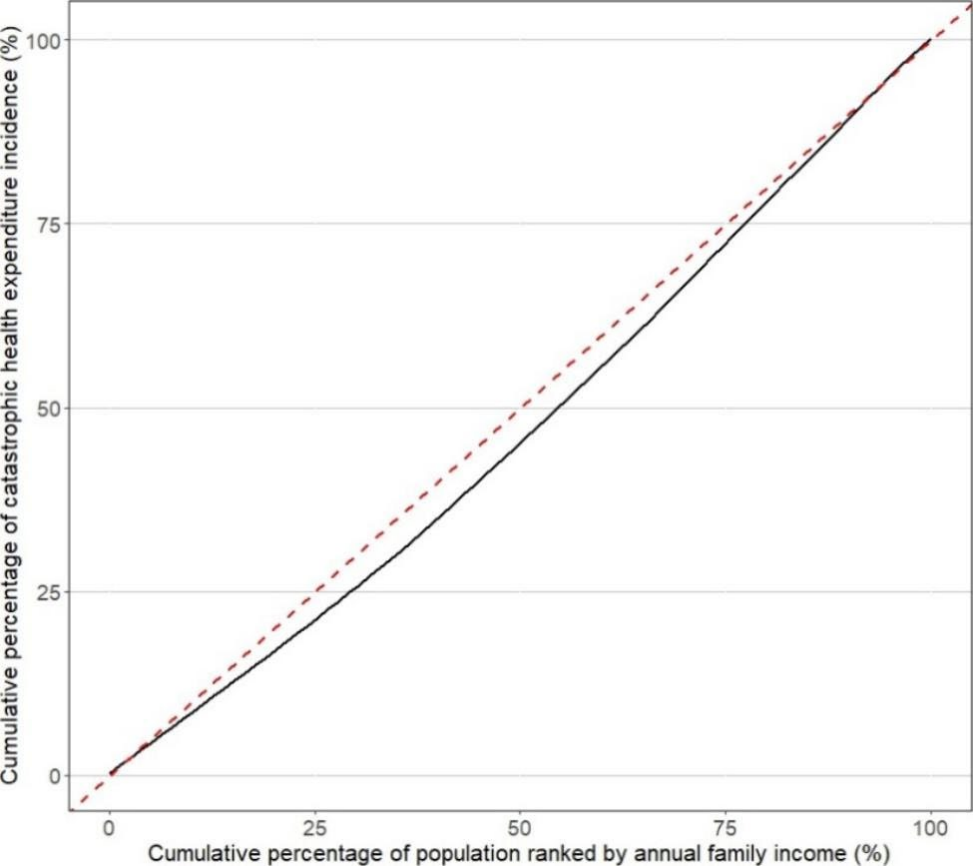
Lorenz curve of catastrophic health expenditure incidence. Notes: The Lorenz curve of catastrophic health expenditure incidence lay below the equal line with a concentration index of 0.059, which revealed that catastrophic health expenditure was concentrated in people with higher socioeconomic status

As depicted in Fig. 2, the Lorenz curve of CHE incidence was under the equal line with a concentration index of 0.059, which indicated that CHE events were more concentrated among people with higher socioeconomic status (pro-rich).

### Association of multimorbidity with the risk of CHE

Both univariate and multivariate models revealed that a higher number of NCDs was significantly associated with an increased risk of CHE (Table 3 and Table S3). In the unadjusted model, the likelihood of CHE increased by 20% for each additional NCD (crude HR = 1.20, 95% CI: 1.17–1.23). In the fully adjusted model, an additional NCD led to a 19% increased risk of CHE (aHR = 1.19, 95% CI: 1.16–1.22).

**Table 3:** Results of univariate and multivariate Cox proportional hazard models, and sensitive analyses

Model		HR (95% CI)	p value
Univariate model		1.20 (1.17, 1.23)	< 0.001
Multivariate model ^a^		1.19 (1.16, 1.22)	< 0.001
Sensitive analyses	model 1^b^	1.20 (1.16, 1.24)	< 0.001
	model 2^c^	1.17 (1.14, 1.20)	< 0.001
	model 3^d^	1.18 (1.15, 1.21)	< 0.001

Notes: HR: hazard ratio; CI: confident interval

a: Model adjusted for gender, age group, marital status, education, insurance, outpatient services, inpatient services, residence, family size, family economic level, and socioeconomic development level

b: Based on the multivariate model, in this model, the definition of CHE (household-medical OOP expenditure exceeding 45% household total expenditure net of food expenditure) was replaced as household-medical OOP expenditure exceeding 25% of total household expenditure

c: Based on the multivariate model, in this model, we changed the original selected main respondents in households with 2 eligible respondents to the 5270 excluded participants

d: Based on the multivariate model, in this model, we used the nighttime light level to indicate socioeconomic development rather than gross regional products

In three sensitivity analyses, the significant association between multimorbidity and CHE incidence did not change when the definition of CHE was replaced as household-medical OOP expenditure exceeding 25% of total household expenditure by the WHO definition; or when the original selected main respondents in households with 2 eligible respondents were changed to the 5270 excluded ones; or when the socioeconomic development level was indicated by nighttime light level rather than GRP (Table 3).

**Fig. 3:**
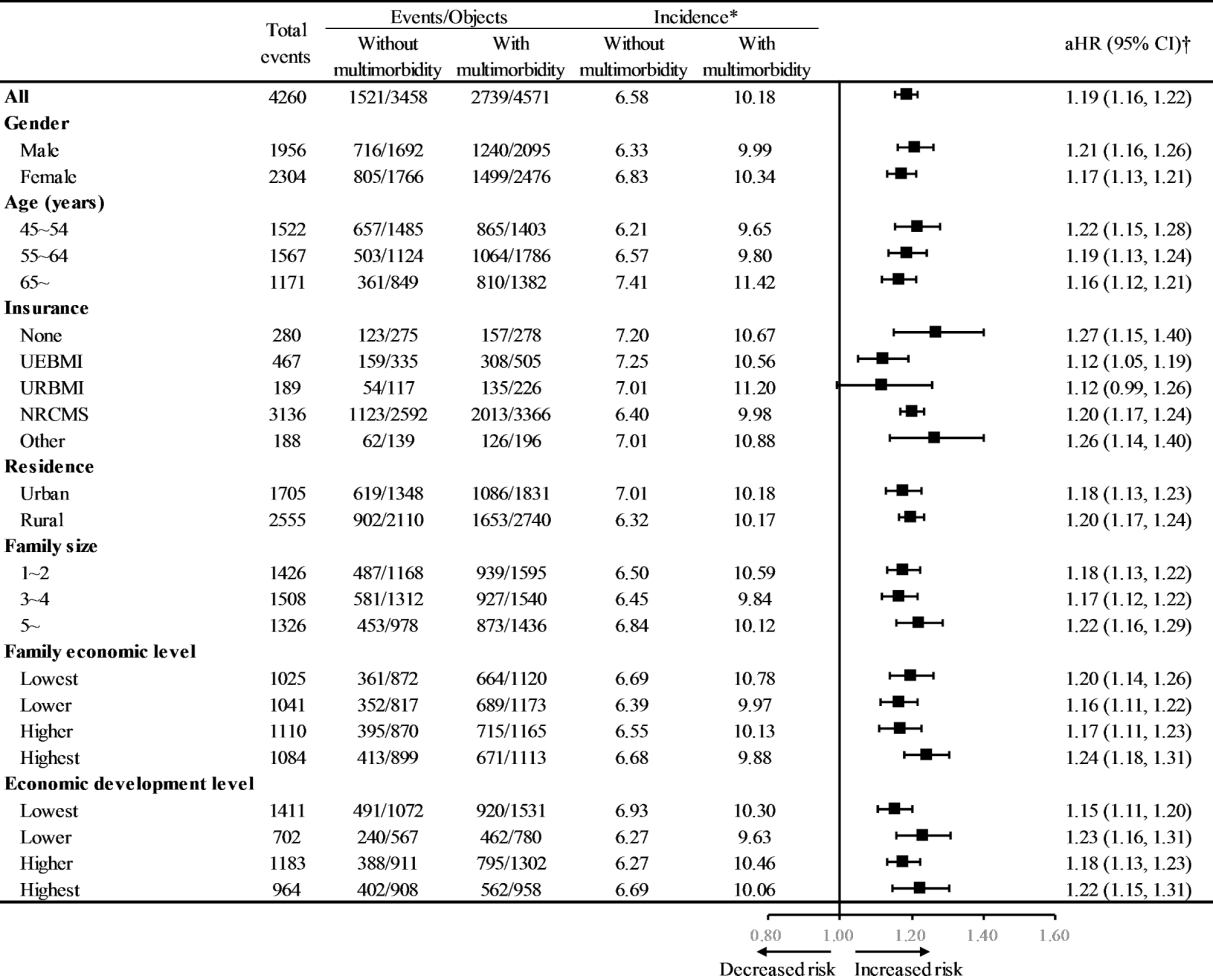
Subgroup analyses of the association between multimorbidity and risk of catastrophic health expenditure. Notes: aHR: adjusted hazard ratio; CI: confident interval; UEBMI: urban employee basic medical insurance; URBMI: urban resident basic medical insurance; NRCMS: new rural cooperative medical scheme. *Per 1000 person-months; † estimation of association between number of NCDs and risk of catastrophic health expenditure

In subgroup analyses, except for household heads with URBMI, the negative effect of multimorbidity on CHE incidence did not appear to be modified by baseline characteristics, including gender, age, family size, residence, family economic level, or economic development level (all p values < 0.05; Fig. 3).

## Discussion

To our knowledge, this study is the first to explore and quantify the effect of multimorbidity on long-term CHE through a cohort study design. We found that multimorbidity was common among Chinese people aged 45 years and above, family economic level and socioeconomic development level were related to multimorbidity, CHE events were more concentrated among people with higher socioeconomic status, and multimorbidity was associated with an increased risk of long-term CHE incidence.

This study showed that the weighted prevalence of multimorbidity among middle-aged and older adults in China was 44.3% in 2011, which was lower than that in 3 other studies that also used data from CHARLS [12, 13, 23]. The gaps between our results and their results can be explained by differences in the included survey year and population age structure: the prevalence of multimorbidity was reported as 52.2% in 2011 (people aged ≥ 45 years with data on physical activity), 63.4% in 2015 (people aged ≥ 45 years), and 65.6% in 2018 (people aged ≥ 60 years) [12, 13, 23]. An upward trend in multimorbidity prevalence appeared with increasing age or year through data from CHARLS. Given that an NCD might not be resolved, the probability of multimorbid conditions increases with age.

We found that people with higher family economic level had a lower multimorbidity prevalence than those with lowest family economic level, and participants with lower and highest economic development levels were also less likely to have multimorbidity. People with lower socioeconomic status had higher multimorbidity prevalence, which was similar to the findings of other studies despite various indicators for defining socioeconomic status [24, 25]. People with lower socioeconomic status may have poor health literacy and unhealthy lifestyles (such as smoking and unhealthy diets), increasing the risk of NCDs and further resulting in multimorbidity [26, 27]. A systematic review revealed that people in lower socioeconomic groups had a significantly higher prevalence of tobacco and alcohol use, also they consumed less fruit, vegetables, fish and other healthy foods than people in high socioeconomic groups [26]. Additionally, people with low socioeconomic level might have less use of medical care. Zhao et al. [15] found that people in the highest expenditure group had a 20% increased number of outpatient visits than people in the lowest expenditure group. Another national study in Korea also demonstrated that the highest socioeconomic group had 58% higher healthcare utilization than other socioeconomic groups [28]. In view of this, more proactive interventions such as health education might be directed towards people in low socioeconomic groups to strengthen the prevention and management of multimorbidity.

From the analysis at the household level, we found that the percentages of households with multimorbidy that used outpatient care and inpatient care were 17.9% and 8.3%, respectively, which were almost twice those of households without multimorbidity (9.2% for outpatient care and 3.6% for inpatient care). A couple of studies drew the conclusions that because of deteriorating health conditions, multimorbidity accounted for more consultations in primary care, more frequent outpatient visits and longer lengths of inpatient stays [29–31]. Notably, 82.1% of participants with multimorbidity did not make use of outpatient care in the last month in our study. Because the regular management of NCDs in primary health care in China allows doctors to prescribe more than one month worth of drugs (i.e., prescription refills) for patients with stable chronic conditions [32, 33], the participantis included in our study might have visited clinics before the investigation month or planned to go to primary health insitutions after the investigation month. In addition, some individuals were unable to seek medical care for financial reasons. Insufficient treatment directly contributes to poor control of multimorbidity, leading to more severe health outcomes, which might require high medical costs to both households and society. To improve patients’ conditions and avoid financial hardship, concerted efforts from related health institutions could be made to increase patients’ rational healthcare utilization.

In addition, we found that CHE events were more concentrated among people with higher socioeconomic status, which was consistent with the findings of some studies but contrary to the findings of others [34, 35]. Given the definition of CHE, it can be summarized that both OOP medical expenditure and family financial capacity can determine the onset of CHE. Therefore, on the one hand, as shown in some studies, people with lower socioeconomic status may be more likely to incur CHE with lower income for calculating CHE. On the other hand, people with higher socioeconomic status might seek health services more frequently without worrying about medical expenditure, contributing to a lager molecule for calculating CHE. Several studies revealed that both health services distribution and utilization were concentrated among people with higher socioeconomic status [4, 36]. These people were prone to overuse high-quality tertiary hospitals with poor quality primary health care, which are distributed mostly in urban areas and require higher costs with lower reimbursement ratios [4]. Notably, a series of policies in China for decreasing the financial risk of health care were introduced with emphasis on rural populations, most with lower socioeconomic status, such as catastrophic health insurance, medical assistance, and basic life assistance [5, 37].

Our results also showed that the risk of long-term household CHE events was 19% higher for each additional NCD, which was consistent with other cross-sectional studies [14, 15]. Due to incurability, multimorbidity requires more long-term health care of reasonable quality (more frequent outpatient or inpatient visits and longer length of inpatient stays) for both primary and secondary health care services, which directly increases medical expenditure [15, 38, 39]. Considering that the early onset of NCDs increases the possibility for multimorbidity in later life, lifecycle management of multimorbidity for reducing CHE should be prioritized, including integrated activities for primary prevention of NCDs at early ages, timely control of the early state of NCDs, and efficient treatment for each NCD to delay the progression to disability and death.

The first strength of this study was its population-based cohort design, which provided more powerful evidence about the association of multimorbidity with CHE than current published cross-sectional studies [14–17]. Second, our study included more information because it used all wave data (2011–2018) from CHARLS with a longer time span than other studies that used only 1 wave of data [14–17]. Third, we focused on the socioeconomic inequities of CHE incidence in addition to the association of multimorbidity with CHE, which introduced new concerns and distinct emphasis on health policies among different populations. However, there are still several limitations in our study. First, survivor bias may exist in our cohort study because some households that had CHE at baseline were excluded, which might underestimate the CHE incidence, as the remaining sample might be less likely to have CHE. Second, data for measuring CHE, including medical expenditures, total expenditures, and food expenditures, were obtained from oral answers, which might lead to recall bias. Third, although the judgement of multimorbidity was conducted by asking participants if whether they had doctor-diagnosed NCDs, there might be inconsistencies with the real conditions because of different diagnostic procedures between doctors.

## Conclusions

Approximately half of middle-aged and older adults in China had multimorbidity, and the risk of long-term household CHE events was 19% higher for each additional NCD. Integrated management and interventions for preventing multimorbidity could be intensified, especially in older populations with low socioeconomic status. In addition, concerted efforts to increase patients’ rational healthcare utilization, provide high-quality primary care, and strengthen medical security for people with high socioeconomic status might be made to advance progress towards UHC in China.

## Electronic supplementary material

Below is the link to the electronic supplementary material.

Supplementary Material 1

## Data Availability

All waves’ data from the CHARLS can be downloaded from the CHARLS official website (http://charls.pku.edu.cn/).

## Abbreviations

SDGs: The Sustainable Development Goals
UHC: Universal health coverage
WHO: The World Health Organization
CHE: Catastrophic health expenditure
UEBMI: The Urban Employee Basic Medical Insurance
NRCMS: The New Rural Cooperative Medical Scheme
URBMI: The Urban Resident Basic Medical Insurance
NCDs: Non-communicable diseases
CHARLS: The China Health and Retirement Longitudinal Study
OOP: Out-of-pocket
C: Concentration index
aORs: Adjusted odds ratios
CIs: Confident intervals
aHRs: Adjusted hazard ratios
GRP: Gross regional product
