# Supplementary material for: Associations among socioeconomic status, multimorbidity of non-communicable diseases, and the risk of household catastrophic health expenditure in China: a population-based cohort study
Source: BMC Health Serv Res. 2023 Apr 26;23:403. doi: 10.1186/s12913-023-09391-x (PMC10131349; doi:10.1186/s12913-023-09391-x)
Supplement: Supplementary file 1 — Supplementary Material 1 [file 12913_2023_9391_MOESM1_ESM.docx]

**Table S1: Weighted and unweighted percentage of baseline characteristics for 17182 eligible participants**

| **Baseline characteristics** | **Number**  **(N=17182)** | **Percentage** | |
| --- | --- | --- | --- |
|  |  | **Unweighted** | **Weighted** |
| **Multimorbidity** |  |  |  |
| No | 9430 | 54.9 | 55.7 |
| Yes | 7752 | 45.1 | 44.3 |
| **Gender** |  |  |  |
| Male | 8364 | 48.7 | 48.4 |
| Female | 8818 | 51.3 | 51.6 |
| **Age** |  |  |  |
| 45~54 | 6020 | 35.0 | 35.9 |
| 55~64 | 6433 | 37.4 | 35.3 |
| 65~ | 4729 | 27.5 | 28.8 |
| **Marital status** |  |  |  |
| Married | 14948 | 87.0 | 85.2 |
| Other | 2234 | 13.0 | 14.8 |
| **Education** |  |  |  |
| Illiterate/semiliterate | 7814 | 45.5 | 42.7 |
| Primary school | 3617 | 21.1 | 20.6 |
| Middle school | 3545 | 20.6 | 21.2 |
| High school and above | 2206 | 12.8 | 15.5 |
| **Insurance** |  |  |  |
| None | 1154 | 6.7 | 7.2 |
| UEBMI | 1892 | 11.0 | 15.6 |
| URBMI | 784 | 4.6 | 5.9 |
| NRCMS | 12614 | 73.4 | 66.2 |
| Other | 738 | 4.3 | 5.0 |
| **BMI** |  |  |  |
| Normal | 10849 | 63.1 | 64.7 |
| Underweight | 966 | 5.6 | 5.2 |
| Overweight | 3892 | 22.7 | 21.9 |
| Obesity | 1475 | 8.6 | 8.2 |
| **Residence** |  |  |  |
| Urban | 6921 | 40.3 | 49.8 |
| Rural | 10261 | 59.7 | 50.2 |
| **Family size** |  |  |  |
| 1~2 | 6194 | 36.0 | 33.8 |
| 3~4 | 5886 | 34.3 | 34.9 |
| 5~ | 5102 | 29.7 | 31.3 |
| **Family economic level** |  |  |  |
| Lowest | 4207 | 24.5 | 22.5 |
| Lower | 4315 | 25.1 | 22.1 |
| Higher | 4341 | 25.3 | 25.7 |
| Highest | 4319 | 25.1 | 29.7 |
| **Economic development level** |  |  |  |
| Lowest | 5398 | 31.4 | 29.2 |
| Lower | 2924 | 17.0 | 15.3 |
| Higher | 4727 | 27.5 | 26.0 |
| Highest | 4133 | 24.1 | 29.5 |

Notes: UEBMI: urban employee basic medical insurance; URBMI: urban resident basic medical insurance; NRCMS: new rural cooperative medical scheme; BMI: body mass index.

**Table S2: Weighted and unweighted percentage of baseline characteristics for 8029 eligible households**

| **Baseline characteristics** | **Number** | |  | **Unweighted percentage** | |  | **Weighted percentage** | |
| --- | --- | --- | --- | --- | --- | --- | --- | --- |
|  | **Without multimorbidity** | **With multimorbidity** |  | **Without multimorbidity** | **With multimorbidity** |  | **Without multimorbidity** | **With multimorbidity** |
| Total | **3458** | **4571** |  | 100.0 | 100.0 |  | 100.0 | 100.0 |
| **Gender** |  |  |  |  |  |  |  |  |
| Male | 1692 | 2095 |  | 48.9 | 45.8 |  | 48.5 | 46.6 |
| Female | 1766 | 2476 |  | 51.1 | 54.2 |  | 51.5 | 53.4 |
| **Age** |  |  |  |  |  |  |  |  |
| 45~54 | 1485 | 1403 |  | 42.9 | 30.7 |  | 44.6 | 30.3 |
| 55~64 | 1124 | 1786 |  | 32.5 | 39.1 |  | 31.5 | 39.1 |
| 65~ | 849 | 1382 |  | 24.6 | 30.2 |  | 23.9 | 30.6 |
| **Marital status** |  |  |  |  |  |  |  |  |
| Married | 2586 | 3747 |  | 74.8 | 82.0 |  | 75.3 | 81.8 |
| Other | 872 | 824 |  | 25.2 | 18.0 |  | 24.7 | 18.2 |
| **Education** |  |  |  |  |  |  |  |  |
| Illiterate/semiliterate | 1567 | 2181 |  | 45.3 | 47.7 |  | 42.2 | 43.8 |
| Primary school | 698 | 967 |  | 20.2 | 21.2 |  | 20.9 | 21.7 |
| Middle school | 728 | 886 |  | 21.1 | 19.4 |  | 20.8 | 19.9 |
| High school and above | 465 | 537 |  | 13.4 | 11.7 |  | 16.1 | 14.5 |
| **Insurance** |  |  |  |  |  |  |  |  |
| None | 275 | 278 |  | 8.0 | 6.1 |  | 8.9 | 6.5 |
| UEBMI | 335 | 505 |  | 9.7 | 11.0 |  | 12.7 | 15.4 |
| URBMI | 117 | 226 |  | 3.4 | 4.9 |  | 4.1 | 6.7 |
| NRCMS | 2592 | 3366 |  | 75.0 | 73.6 |  | 69.7 | 66.4 |
| Other | 139 | 196 |  | 4.0 | 4.3 |  | 4.6 | 5.0 |
| **Outpatient care** |  |  |  |  |  |  |  |  |
| No | 3140 | 3755 |  | 90.8 | 82.1 |  | 89.0 | 82.2 |
| Yes | 318 | 816 |  | 9.2 | 17.9 |  | 11.0 | 17.8 |
| **Inpatient care** |  |  |  |  |  |  |  |  |
| No | 3332 | 4192 |  | 96.4 | 91.7 |  | 95.8 | 91.9 |
| Yes | 126 | 379 |  | 3.6 | 8.3 |  | 4.2 | 8.1 |
| **BMI** |  |  |  |  |  |  |  |  |
| Normal | 2345 | 2783 |  | 67.8 | 60.9 |  | 67.7 | 62.3 |
| Underweight | 203 | 264 |  | 5.9 | 5.8 |  | 5.2 | 5.4 |
| Overweight | 678 | 1066 |  | 19.6 | 23.3 |  | 20.6 | 22.5 |
| Obesity | 232 | 458 |  | 6.7 | 10.0 |  | 6.6 | 9.9 |
| **Residence** |  |  |  |  |  |  |  |  |
| Urban | 1348 | 1831 |  | 39.0 | 40.1 |  | 47.3 | 49.0 |
| Rural | 2110 | 2740 |  | 61.0 | 59.9 |  | 52.7 | 51.0 |
| **Family size** |  |  |  |  |  |  |  |  |
| 1~2 | 1168 | 1595 |  | 33.8 | 34.9 |  | 33.5 | 35.5 |
| 3~4 | 1312 | 1540 |  | 37.9 | 33.7 |  | 38.9 | 32.5 |
| 5~ | 978 | 1436 |  | 28.3 | 31.4 |  | 27.7 | 32.0 |
| **Family economic level** |  |  |  |  |  |  |  |  |
| Lowest | 872 | 1120 |  | 25.2 | 24.5 |  | 24.5 | 22.1 |
| Lower | 817 | 1173 |  | 23.6 | 25.7 |  | 21.3 | 23.4 |
| Higher | 870 | 1165 |  | 25.2 | 25.5 |  | 25.2 | 26.7 |
| Highest | 899 | 1113 |  | 26.0 | 24.3 |  | 29.0 | 27.9 |
| **Economic development level** | |  |  |  |  |  |  |  |
| Lowest | 1072 | 1531 |  | 31.0 | 33.5 |  | 29.6 | 30.8 |
| Lower | 567 | 780 |  | 16.4 | 17.1 |  | 14.6 | 15.8 |
| Higher | 911 | 1302 |  | 26.3 | 28.5 |  | 24.1 | 26.6 |
| Highest | 908 | 958 |  | 26.3 | 21.0 |  | 31.7 | 26.9 |

Notes: UEBMI: urban employee basic medical insurance; URBMI: urban resident basic medical insurance; NRCMS: new rural cooperative medical scheme; BMI: body mass index.

**Table S3: Association of multimorbidity and risk of household catastrophic health expenditure**

|  | **Total**  **(Events/Objects)** | **Without multimorbidity**  **(Events/Objects)** | **With multimorbidity**  **(Events/Objects)** | **aHR (95%CI)** | ***p-*value** |
| --- | --- | --- | --- | --- | --- |
| **Total** | 4260/8029 | 1521/3458 | 2739/4571 |  |  |
| **Number of NCDs** | — | — | — | **1.19 (1.16, 1.22)** | **<0.001^*^** |
| **Gender** |  |  |  |  |  |
| Male | 1956/3787 | 716/1692 | 1240/2095 | 1.00 (ref.) | — |
| Female | 2304/4242 | 805/1766 | 1499/2476 | 1.07 (0.99, 1.16) | 0.073 |
| **Age** |  |  |  |  |  |
| 45~54 | 1522/2888 | 657/1485 | 865/1403 | 1.00 (ref.) | — |
| 55~64 | 1567/2910 | 503/1124 | 1064/1786 | 1.16 (1.07, 1.25) | <0.001^*^ |
| 65~ | 1171/2231 | 361/849 | 810/1382 | 1.06 (0.99, 1.13) | 0.086 |
| **Marital status** |  |  |  |  |  |
| Married | 3553/6333 | 1238/2586 | 2315/3747 | 1.00 (ref.) | — |
| Other | 707/1696 | 283/872 | 424/824 | 0.68 (0.61, 0.75) | <0.001^*^ |
| **Education** |  |  |  |  |  |
| Illiterate/semiliterate | 1936/3748 | 664/1567 | 1272/2181 | 1.00 (ref.) | — |
| Primary school | 905/1665 | 321/698 | 584/967 | 1.03 (0.91, 1.16) | 0.637 |
| Middle school | 870/1614 | 332/728 | 538/886 | 0.99 (0.90, 1.08) | 0.788 |
| High school and above | 549/1002 | 204/465 | 345/537 | 1.08 (0.99, 1.18) | 0.072 |
| **Insurance** |  |  |  |  |  |
| None | 280/553 | 123/275 | 157/278 | 1.00 (ref.) | — |
| UEBMI | 467/840 | 159/335 | 308/505 | 0.87 (0.66, 1.13) | 0.293 |
| URBMI | 189/343 | 54/117 | 135/226 | 1.08 (0.78, 1.49) | 0.634 |
| NRCMS | 3136/5958 | 1123/2592 | 2013/3366 | 0.80 (0.66, 0.98) | 0.032^*^ |
| Other | 188/335 | 62/139 | 126/196 | 0.89 (0.67, 1.19) | 0.429 |
| **Outpatient care** |  |  |  |  |  |
| No | 3577/6895 | 1369/3140 | 2208/3755 | 1.00 (ref.) | — |
| Yes | 683/1134 | 152/318 | 531/816 | 1.12 (1.00, 1.24) | 0.040^*^ |
| **Inpatient care** |  |  |  |  |  |
| No | 3937/7524 | 1461/3332 | 2476/4192 | 1.00 (ref.) | — |
| Yes | 323/505 | 60/126 | 263/379 | 1.17 (1.02, 1.35) | 0.026^*^ |
| **BMI** |  |  |  |  |  |
| Normal | 2662/5128 | 1013/2345 | 1649/2783 | 1.00 (ref.) | — |
| Underweight | 224/467 | 89/203 | 135/264 | 0.90 (0.77, 1.05) | 0.191 |
| Overweight | 983/1744 | 313/678 | 670/1066 | 0.91 (0.84, 0.99) | 0.045^*^ |
| Obesity | 391/690 | 106/232 | 285/458 | 0.89 (0.78, 1.00) | 0.057 |
| **Residence** |  |  |  |  |  |
| Urban | 1705/3179 | 619/1348 | 1086/1831 | 1.00 (ref.) | — |
| Rural | 2555/4850 | 902/2110 | 1653/2740 | 0.87 (0.80, 0.94) | 0.001^*^ |
| **Family size** |  |  |  |  |  |
| 1~2 | 1426/2763 | 487/1168 | 939/1595 | 1.00 (ref.) | — |
| 3~4 | 1508/2852 | 581/1312 | 927/1540 | 1.07 (0.99, 1.15) | 0.066 |
| 5~ | 1326/2414 | 453/978 | 873/1436 | 1.02 (0.95, 1.08) | 0.615 |
| **Family economic level** |  |  |  |  |  |
| Lowest | 1025/1992 | 361/872 | 664/1120 | 1.00 (ref.) | — |
| Lower | 1041/1990 | 352/817 | 689/1173 | 0.90 (0.82, 0.98) | 0.012^*^ |
| Higher | 1110/2035 | 395/870 | 715/1165 | 1.04 (0.96, 1.11) | 0.340 |
| Highest | 1084/2012 | 413/899 | 671/1113 | 0.96 (0.89, 1.03) | 0.267 |
| **Economic development level** |  |  |  |  |  |
| Lowest | 1411/2603 | 491/1072 | 920/1531 | 1.00 (ref.) | — |
| Lower | 702/1347 | 240/567 | 462/780 | 1.03 (0.95, 1.11) | 0.545 |
| Higher | 1183/2213 | 388/911 | 795/1302 | 1.16 (1.07, 1.25) | <0.001^*^ |
| Highest | 964/1866 | 402/908 | 562/958 | 1.01 (0.94, 1.09) | 0.726 |

Notes: aHR: adjusted hazard ratio; CI: confidence interval. UEBMI: urban employee basic medical insurance; URBMI: urban resident basic medical insurance; NRCMS: new rural cooperative medical scheme; BMI: body mass index.

*p<0.05
